# Analysis and mapping of harm reduction research in the context of injectable drug use: identifying research hotspots, gaps and future directions

**DOI:** 10.1186/s12954-024-01048-0

**Published:** 2024-07-10

**Authors:** Waleed M. Sweileh

**Affiliations:** Department of Physiology and Pharmacology/Toxicology, Division of Biomedical Sciences, College of Medicine and Health Sciences, An-Najah National University, Nablus, Palestine

**Keywords:** Injectable drug use, Harm reduction research, Literature analysis, Research hotspots, Knowledge gaps

## Abstract

**Background:**

Harm reduction is a crucial approach in addressing the multifaceted challenges of injectable drug use. This paper presents an analysis and mapping of the existing literature on harm reduction research in the context of injectable drug use. By reviewing a comprehensive set of scholarly articles, this study identifies research hotspots, knowledge gaps, and future directions in the field. The findings provide valuable insights for researchers, policymakers, and practitioners to guide future research efforts and inform evidence-based harm reduction interventions.

**Methods:**

Data for the study was obtained from the Scopus database, using keywords and phrases related to harm reduction and injectable drug use. Validation methods were employed to verify the accuracy and comprehensiveness of the search strategy. Data analysis involved identifying growth patterns, key contributors, mapping frequent terms, identifying research hotspots, and identifying emerging research directions.

**Results:**

A total of 971 articles were found, with a notable increase from 2015 to 2022. The *International Journal of Drug Policy* (*n* = 172, 17.7%) and the *Harm Reduction Journal* (*n* = 104, 10.7%) were the most prolific journals, and the United States (*n* = 558, 57.5%) had the highest number of publications. The *Johns Hopkins University* (*n* = 80, 8.5%) was the most prolific institution. Mapping of frequent author keywords revealed the main keywords, including harm reduction, HIV, hepatitis C, and opioid overdose. The highly cited articles cover a broad time span and focus on topics like naloxone distribution, HIV and hepatitis C transmission, while recent articles concentrate on emerging issues such as the impact of the COVID-19 pandemic, fentanyl-related concerns, stigma reduction, and needle and syringe programs. Both sets of articles share a common focus on harm reduction strategies, but recent publications highlight current challenges and developments in the field.

**Conclusions:**

This study provides insights into research landscape on harm reduction in injectable drug use. Research is concentrated in high-income countries, emphasizing the need for more research in low- and middle-income countries. Recent publications focus on emerging challenges like COVID-19 and fentanyl. Research gaps highlight the need for studies in diverse populations, social determinants, program evaluation, and implementation strategies to enhance harm reduction interventions.

**Supplementary Information:**

The online version contains supplementary material available at 10.1186/s12954-024-01048-0.

## Background

Substance use disorder (SUD) is a condition in which the consumption of one or more substances results in clinically significant impairment or distress. This disorder is marked by the recurrent use of alcohol and/or drugs, leading to serious health problems, disability, and an inability to fulfill major responsibilities at work, school, or home [1, 2]. The excessive and repeated intake of these substances brings about detrimental physical, psychological, and social effects. SUD imposes a substantial global burden, affecting individuals, families, and communities by compromising health, social relationships, and economic stability [3–5]. Among the various substances abused, injectable drug use (IDU) poses the most severe health and overdose risks [6]. Injectable drugs, administered directly into the bloodstream via needle and syringe, include illicit substances like heroin and cocaine, as well as certain prescription medications such as opioids [7]. The prevalence of IDU within the broader spectrum of SUD varies across regions and populations. According to the World Drug Report 2020 by the United Nations Office on Drugs and Crime (UNODC), approximately 10.5 million people globally engage in drug injection [8].

Harm reduction and prevention strategies play a crucial role in addressing IDU and its associated harms [9, 10]. One significant harm is the risk of overdose, which can be addressed through various strategies. For instance, distributing naloxone, an opioid overdose reversal medication, to individuals who use opioids can save lives [9, 11, 12]. Harm reduction also focuses on preventing the transmission of blood-borne diseases like HIV and hepatitis by providing clean needles and syringes through needle and syringe programs. These initiatives help reduce the spread of infections among people who inject drugs. Harm reduction initiatives and prevention strategies work to mitigate negative health consequences of IDU by promoting safer practices and reducing stigmatization. By providing individuals with accurate information and education about safer drug use practices, harm reduction programs help minimize the risks associated with IDU. Additionally, supporting individuals in their recovery journey through counseling, peer support, and access to treatment options can positively impact their lives and reduce the social and economic burdens of IDU [11, 13]. Harm reduction and prevention efforts focus on ensuring individuals have access to evidence-based interventions, support services, and treatment options. This includes providing opioid agonist treatment, which involves substituting illicit opioids with prescribed medications such as methadone or buprenorphine [9, 14, 15]. Opioid agonist treatment helps individuals manage their substance use disorder, reduce cravings, and improve their overall well-being [12, 13, 16]. Harm reduction and prevention strategies have broader implications for public health and community safety. By preventing the spread of diseases like HIV and hepatitis, harm reduction initiatives contribute to the overall well-being of communities. Furthermore, these approaches can help reduce drug-related crime rates. By shifting the focus from punitive measures to public health-oriented strategies, such as outreach programs and harm reduction services, communities can create safer environments and reduce the negative impacts of drug-related criminal activities [17].

The research landscape refers to the overall state of scientific knowledge, studies, and research activities in a specific field or area of inquiry. It encompasses the existing literature, research gaps, methodologies, key findings, and emerging trends within the subject of interest [18]. The current study aims to identify current state of scientific literature, identifying gaps, and provide recommendations for future research priorities and directions to reduce harm in the context of IDU. A landscape research analysis on harm reduction of IDU is expected to provide a comprehensive overview of the existing literature, identify research hotspots, and highlight gaps and areas for further research. By analyzing the research landscape, policymakers and funders can gain insights into areas that require increased investment and support. This analysis helps ensure that resources are allocated efficiently to address priority research questions and areas of need. Research landscape analysis allows researchers and organizations to identify potential collaborators and foster interdisciplinary collaborations. By understanding the existing research networks and collaborations, stakeholders can work together to address complex challenges in harm reduction among individuals who inject drugs.

## Methods

### Data source

The primary data source for the current study was Scopus, a comprehensive bibliographic database that includes a wide range of scholarly publications across various disciplines (www.scopus.com).

### Search strategy

A search was conducted using relevant keywords and phrases related to harm reduction and “IDU”. All retrieved articles must include specific terms in their titles to ensure relevance to IDU. These terms were: “opioid*” or “heroin” or “inject* drugs” or (“inject” and “stimulant*”) or “needle drug use” or (“inject” and “opioid”) or (“inject” and “heroin”) or (“inject” and “cocaine”) or (“inject” and “amphetamine”) or (inject* and drug*). This comprehensive set of search terms ensures that the articles retrieved were directly related to the study of opioid use, heroin use, injectable drugs, stimulant injection, and needle drug use. To ensure that these terms were used in the context of injections, the following keywords must be present in the title and/or abstract: inject* or needle* or intravenous or syringe*. Harm reduction is a broad concept and developing a comprehensive search strategy might be difficult. However, in the current study, maximum number of available keywords related to harm reduction, programs, interventions, policies, initiatives were included. To ensure that retrieved articles are relevant to harm reduction, they must include specific terms or phrases in their titles and/or abstracts. these terms are: “collaboration with community organizations” or “family and social network involvement” or “supportive housing” or “employment and vocational support” or “education and awareness” or “policy interventions” or “treatment and rehabilitation” or “media and advertising regulations” or “research and monitoring” or “prevent* blood borne infect*” or “harm reduction” or “harm minimization” or “risk reduction” or “damage reduction” or “health promotion” or “safer use” or “safety-focused approaches” or “minimizing adverse consequences” or “public health approach” or “health-centered interventions” or “prevention of harm” or “behavior-based interventions” or “reduction of negative outcomes” or “mitigation of risks” or “safety strategies” or “needle exchange program*” or “safer injection practice*” or “overdose prevention” or “safe substance use” or “substance use treatment alternatives” or “syringe access program*” or “safer drug use practice*” or “syringe exchange program” or “HIV transmission” or “virus transmission” or “condom use” or (“blood borne infect*” and prevent*) or “needle and syringe program*” or “harm reduction program*” or “needle distribution program*” or “opioid substitution therapy” or “medication-assisted treatment” or “opioid detoxification programs” or “education and awareness campaign*” or “substance abuse prevention campaign*” or “drug education programs” or “public awareness initiatives” or “information campaign*” or “safer sex practices” or “harm reduction in sexual behavior” or “safe sex education” or “condom use promotion” or “barrier method education” or “preventing sexually transmitted” or “harm reduction drug testing” or “naloxone distribution” or “naloxone access programs” or “overdose reversal medication distribution” or “narcan distribution” or “opioid overdose rescue kits” or “supervised consumption facilit*” or “medically supervised drug consumption site*” or “overdose prevention centers” or “supervised injection services” or “street outreach service*” or “mobile harm reduction program*” or “outreach services for people who use drugs” or “harm reduction outreach activities” or “peer support program*” or “peers in recovery programs” or “peer mentoring for substance abuse” or “support networks of individuals with lived experience” or “peer-based harm reduction services” or “overdose prevention education” or “overdose response training” or “naloxone training program*” or “opioid overdose education” or “recognizing and responding to overdose*” or “overdose prevention initiative*” or “access to healthcare service*” or “healthcare for people who use drugs” or “substance abuse treatment access” or “integrated healthcare for substance use disorder*” or “addiction treatment service*” or “medical service* for substance abuser*” or “harm reduction policies and advocacy” or “public health campaign*” or “harm reduction in prison*” or “harm reduction psychotherapy” or “testing and treatment for blood-borne diseases” or “peer education and safer use practice*” or “peer support and peer distribution programs” or “risk mitigation” or “minimizing harm” or “injection safety” or “drug-related harm reduction” or “substance use harm reduction” or “health protection strategies” or “public health approaches to substance abuse” or “drug-related harm prevention” or “safe disposal of drug paraphernalia” or “opioid overdose reversal” or “managed drug consumption site*”. Supplement 1 provides all keywords used in the search string. Boolean operators (e.g., and, or) were used to refine the search and capture relevant articles.

### Inclusion and exclusion criteria

The retrieved articles were screened based on specific inclusion and exclusion criteria. Inclusion criteria encompassed research articles published in peer-reviewed journals, written in English, and published at any time between 1980 and December 31st, 2022. Exclusion criteria involved articles that were not directly related to harm reduction in the context of IDU. For example, articles related to articles related to alcohol, tobacco, or smoking were excluded.

### Validation

#### Absence of false positive results

The top 40 cited articles were thoroughly reviewed to verify their relevance to the field of harm reduction interventions for substance use. Each article was assessed to confirm that it addressed the research topic and contained information pertinent to the study objectives. The absence of false positive results within the top 40 cited articles provided confidence in the accuracy of the search strategy. To further verify the absence of false-positive results, a random sample of 20 articles was screened, and no false positives were found. This sample was chosen by selecting every 10th article after sorting the articles based on the year of publication.

#### Active journals within the field

Scopus provides the names of journals involved in publishing the retrieved articles. Journals that published the highest number of articles (active journals) were examined to assess their relevance and standing within the field of substance use. In the current study, the *International Journal of Drug Policy*, *Harm Reduction Journal*, and *Drug and Alcohol Dependence* were most active within the field indicating that the implemented methodology was abe to identify sources that publish research articles in the area of interest.

#### Active authors within the field

Scopus provides the names of authors with the highest number of publications in a given set of data. Authors with the highest number of publications (active authors) were assessed to ensure that their expertise and research interest were in the field of harm reduction in general. In the current study, Scopus profiles of active authors indicated that the research field and interests of active authors indicated that the search methodology was able to identify key contributors within the field.

#### Comprehensive coverage

The comprehensiveness of the search strategy was tested by conducting a cross-validation with an alternative source. To validate the scope of the search strategy, ten articles were randomly selected from Google Scholar, a commonly used academic search engine, that had the term “harm reduction” and “substance use”. These ten articles were then searched for within the retrieved articles from Scopus. The presence of all ten articles in the retrieved dataset indicated the thoroughness and comprehensiveness of the search strategy.

#### Correlation testing

Based on the search strategy, Scopus provides the list of authors involved in published the retrieved articles. In the current study, Vickerman, P., Des Jarlais D.C., Strathdee, S.A., Maher, L. and others were the most active. For each active author, Scopus denotes the number of articles contributed by each active author to the field. To confirm the validity of the search strategy, we tested the correlation between the numbers of articles provided by Scopus for the top 10 active authors with the number of articles for the same authors counted manually in Google Scholar. In the current study, there was a significant correlation (*p* = 0.019) with a Pearson correlation of 0.83. This indicated high validity and comprehensiveness of the search strategy. This method was adopted from previously published methodology in the field of research landscape analysis [19].

### Data analysis

#### Growth pattern of publications and key contributors to the retrieved articles

They were identified by exporting the retrieved articles from Scopus to Microsoft Excel, sorted, and presented. Key contributors included active authors, countries, journals, and institutions. In the current study, the term “active” refers to the those with highest number of publications.

#### VOSviewer maps

In the current study, three types of maps were created using VOSviewer [20], a software tool for visualizing and analyzing networks. Interpreting VOSviewer maps involves understanding how different elements of academic literature are visually represented. The author-author collaboration network map illustrates the relationships and collaborations between authors within a specific field. Each node represents an author, and the links between nodes indicate collaboration, typically through co-authorship of papers. Larger nodes indicate authors with more publications or greater centrality in the network, suggesting they are key figures or highly productive researchers in the field. Thicker or more numerous links between nodes suggest stronger or more frequent collaborations, while a dense cluster of links implies a tight-knit group of authors who frequently collaborate. Authors who collaborate frequently with each other tend to form clusters, represented by different colors, which may correspond to a subfield or a specific research group within the larger field. Isolated nodes indicate authors without links, suggesting they have not collaborated with others in the dataset or have fewer collaborative ties. The map of most frequent author keywords shows the keywords that authors use most frequently in their publications, helping identify the main topics and trends within the research field. Larger nodes represent more frequently used keywords, indicating prominent topics or themes in the literature. Links between keywords show how often they appear together in the same articles, with stronger or more frequent links suggesting a close relationship between those topics. Keywords that frequently co-occur form clusters, usually represented by different colors, signifying thematic areas or specific research topics within the field. High-density areas on the map indicate heavily researched topics with many publications, often central themes in the field. The map of most frequent terms in titles and abstracts highlights the most common terms found in the titles and abstracts of the publications, providing insights into the primary focus and content of the research. Larger nodes represent terms that appear more frequently in titles and abstracts, highlighting key concepts and focal points of the research. Links between terms show how often they appear together in the same titles or abstracts, with stronger or more frequent links indicating these terms are commonly associated with each other. Terms that frequently co-occur are grouped into clusters, each represented by a different color, revealing conceptual areas or common themes within the research field. Central terms are those that appear frequently and are connected to many other terms, suggesting they are core concepts in the field, while peripheral terms are less frequent and may represent niche or emerging topics. VOSviewer often uses color coding to represent different clusters or groups, and understanding the color legend is essential for interpreting the map accurately. The overlay visualization feature can show temporal trends by mapping the average publication year of items, helping identify emerging trends or shifts in research focus over time. The density visualization highlights areas with a high concentration of items, indicating popular or heavily researched topics. VOSviewer allows users to interact with the map, such as zooming in and out or clicking on nodes for more information, enabling a thorough exploration of details and relationships. By carefully examining these maps, researchers can gain valuable insights into collaboration patterns, research trends, and key topics within their field of interest.

#### Research hotspots identification

To identify research hotspots, the ones with high influence and impact in the field, the top 50 cited articles were screened to identify the top main five research topics.

#### Identification of emerging research directions

To identify the current research topics and potential future research directions, analysis of the recently published 50 articles was conducted.

## Results

### Growth of publications and key contributors to the retrieved articles

**Fig. 1:**
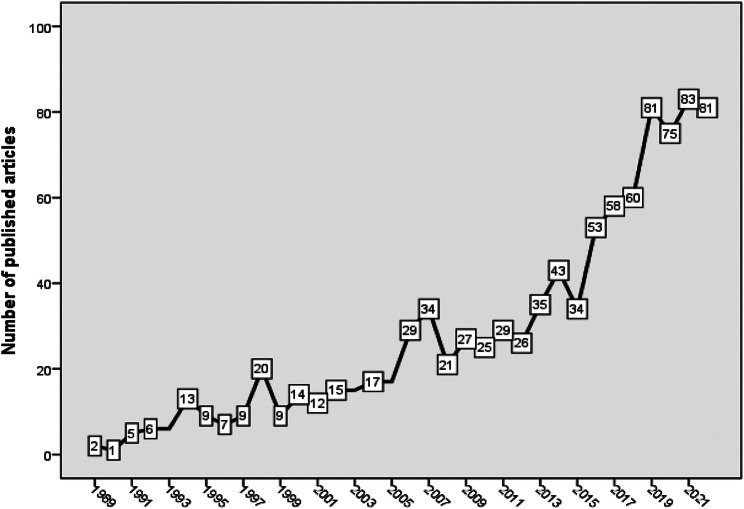
Annual growth of publications on harm reduction research in the context of injectable drug use (1980–2022)

Based on the search strategy outlined in Supplement 1 and flow chart in Supplement 2, a total of 971 articles on harm reduction in the context of IUD were found. Scopus provides categorization of the retrieved articles into different subject areas, based on the scope of each journal. The majority of the retrieved articles were published in journals within the subject area of medicine (*n* = 881, 90.7%), indicating a strong focus on the medical aspects of IDU and harm reduction. Other significant subject areas included social sciences (*n* = 150, 15.4%) and psychology (*n* = 93, 9.6%), highlighting the multidisciplinary nature of research in this field. The number of publications over time showed a slow upward fluctuating increase up to 2014, followed by a steeper increase from 2015 up to 2022 (Fig. 1).

The *International Journal of Drug Policy* emerged as the most prolific journal with 172 (17.7%) articles, followed by *Harm Reduction Journal* (*n* = 104, 10.7%) and *Drug and Alcohol Dependence* (*n* = 38, 3.9%). These three top journals contributed 314 (32.3%) articles to the retrieved literature making them key players in the field. The top 10 active journals were in the field of substance use, drug policy, AIDS, and public health.

**Fig. 2:**
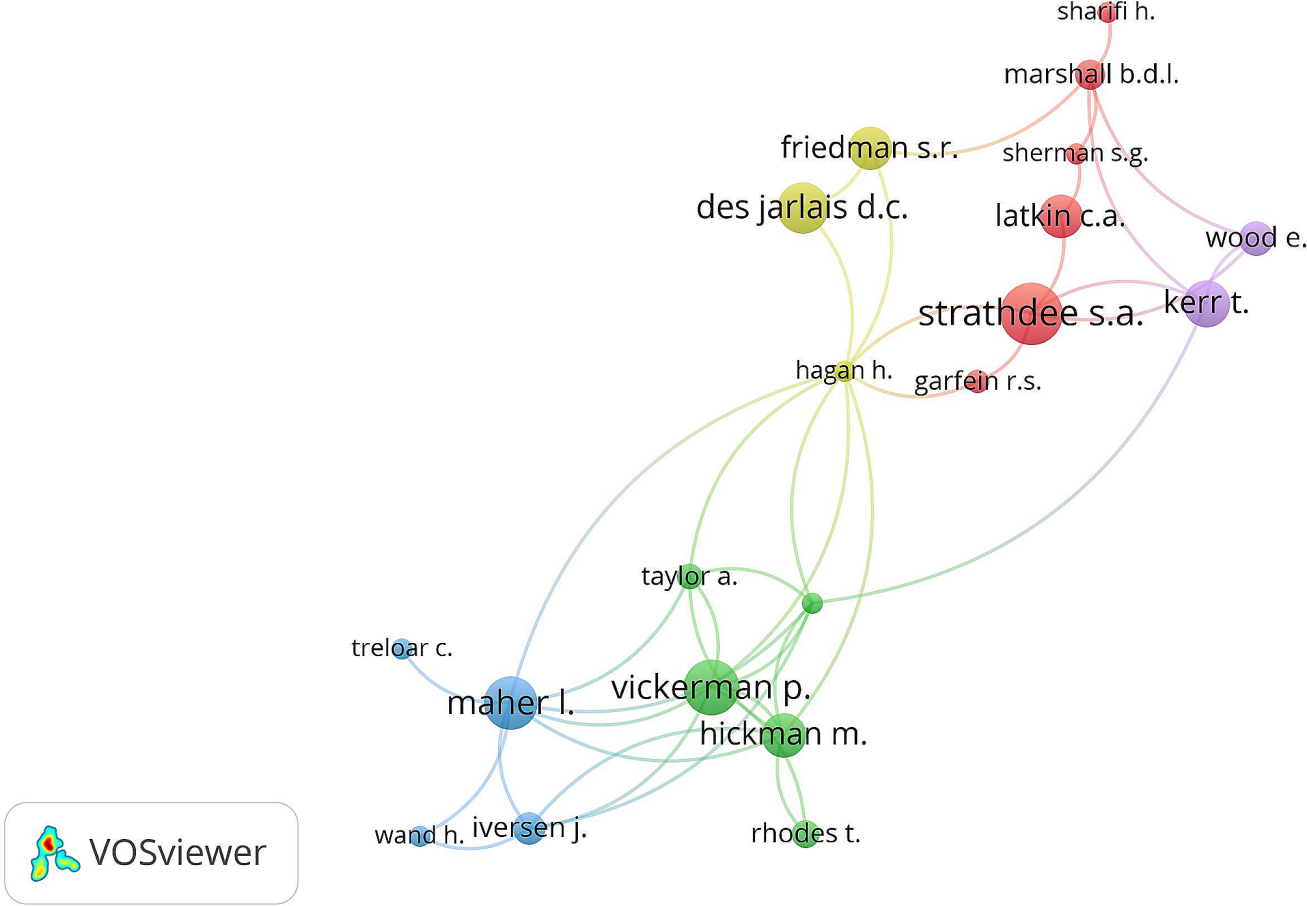
Network visualization map of authors with a minimum contribution of 10 articles and exist in a collaborative research network. The node size of each author is proportional to his/her research contributions. The figure was created by the author using VOSviewer

Scopus provides the country affiliation of each author present in the retrieved articles. Based on this, 83 different country affiliations were found suggesting that scholars from 83 different countries participated in publishing the retrieved articles. The United States, as a country affiliation, had the highest number of publications with 558 articles, accounting for 57.5% of the total. The United Kingdome (UK), Canada, and Australia made significant contributions ranging from 118 to 123 articles for each. However, it’s important to highlight that 41 (49.4%) countries contributed three or fewer articles each, indicating a need for more global research collaboration and participation. Among the institutions, *Johns Hopkins University* ranked as the most prolific with 80 (8.2%) articles, followed by *UNSW Sydney* (*n* = 60, 6.2%), and the *University of British Columbia* (*n* = 51, 5.2%). The retrieved articles involved a total of 3,847 authors, resulting in an average of 4.0 authors per article. Authors with a minimum contribution of 10 articles and exist in a collaborative research network are shown in Fig. 2. The node size of each author is proportional to his/her research contributions. Authors with similar node colors have common research interest. More than one third (*n* = 345, 35.5%) of the published articles were funded by the National Institutes of Health (NIH), highlighting their support and investment in research related to harm reduction interventions.

### Mapping frequent author keywords and frequent terms in titles/abstracts

**Fig. 3:**
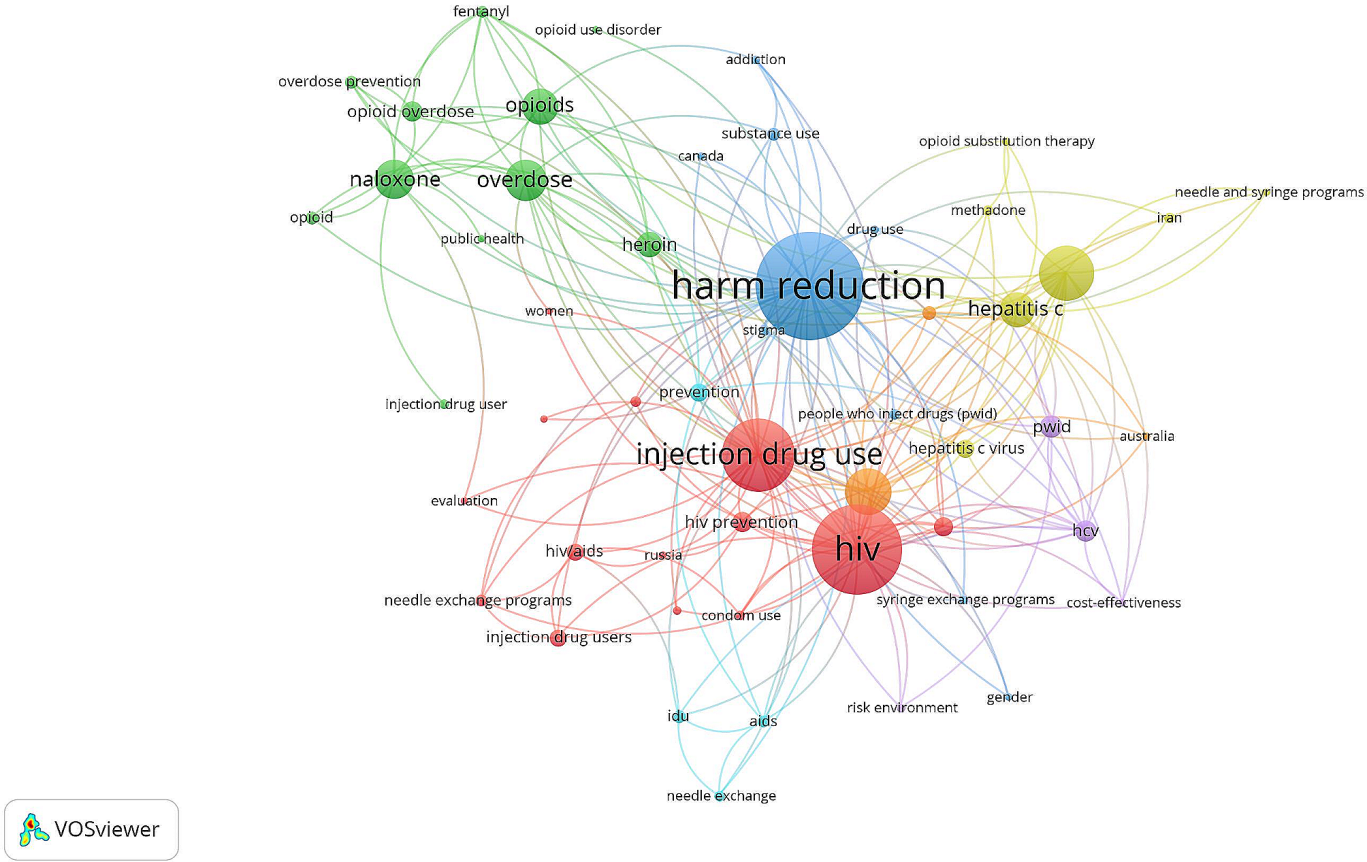
Network visualization map of author keywords that appeared at least 10 times in the collected literature. The map consisted of 52 frequently occurring author keywords. The size of each node in the map corresponds to the frequency of occurrence of the respective keyword in the literature

**Fig. 4:**
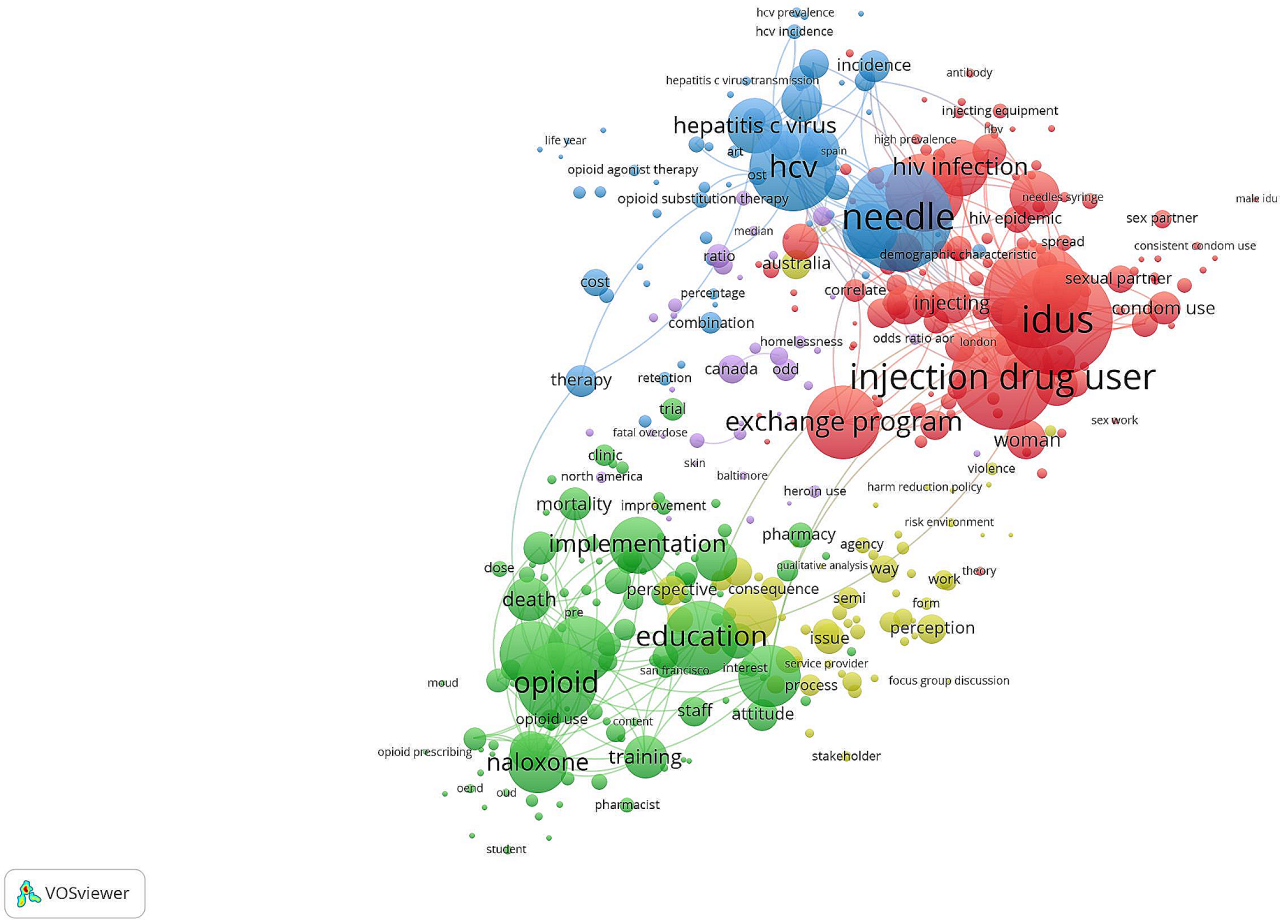
Network visualization map of frequent terms in the titles and abstracts of the retrieved articles. The map shows that the frequent terms were distributed into three major clusters based on node colors. The major research themes were related to harm reduction in the context of HCV (blue cluster), HIV (red cluster), and opioid overdose (green). These represent the main research themes addressed in the retrieved literature

In this study, the VOSviewer technique was utilized to generate a map of author keywords that appeared at least 10 times in the collected literature. The resulting map (Fig. 3) consisted of 52 frequently occurring author keywords. The size of each node in the map corresponds to the frequency of occurrence of the respective keyword in the literature. Among the identified keywords, the ones related to harm reduction and IDU were the most frequent followed by those related to HIV, hepatitis C (HCV), and opioid overdose. VOSviewer visualization technique was also used to map frequent terms in the titles and abstracts of the retrieved articles. The map (Fig. 4) shows the frequent terms distributed into three major clusters based on node colors. The major research themes were related to harm reduction in the context of HCV (blue cluster), HIV (red cluster), and opioid overdose (green). These represent the main research themes addressed in the retrieved literature.

### Research topics in the top 50 cited articles and top 50 recently published articles

Analysis of the top 50 cited articles (Supplement 3) indicated the presence of 10 research topics (Table 1). Based on the title/abstract of the top 50 cited articles, the largest research topic was related to the distribution of naloxone, a medication used to reverse opioid overdoses, and the prevention of opioid overdose deaths. Naloxone distribution programs aim to increase access to naloxone and educate laypersons on its use to save lives in the event of an overdose. This topic directly relates to harm reduction by reducing opioid-related deaths. Analysis of the top 50 recently published articles (Supplement 4) indicated the presence of 9 research topics (Table 2). The largest research topic focused on strategies, interventions, and policies aimed at preventing opioid overdose, including naloxone distribution programs, overdose risk mitigation, overdose prevention training, and opioid overdose counseling. Differences between the nature and content of research topics in the 50 most cited articles and the 50 most recently published articles reflect the differences in research interest and research focus over time. The differences are summarized below:

**Table 1:** Research topics in the top 50 cited articles on harm reduction related to injectable drug use

Research Topic	Brief Description and Relation to Harm Reduction
Naloxone distribution and overdose prevention	Articles related to the distribution of naloxone, a medication used to reverse opioid overdoses, and the prevention of opioid overdose deaths. Naloxone distribution programs aim to increase access to naloxone and educate laypersons on its use to save lives in the event of an overdose. This topic directly relates to harm reduction by reducing opioid-related deaths.
HIV transmission and injection drug use	Articles focusing on the incidence and transmission of HIV among people who inject drugs (PWID). These studies often evaluate the impact of needle and syringe programs, harm reduction interventions, and other prevention strategies on reducing HIV transmission among PWID. This topic addresses harm reduction by targeting the prevention of HIV infection in the context of injection drug use.
Hepatitis C transmission and injection drug use	Articles exploring the transmission and prevention of hepatitis C virus (HCV) among people who inject drugs. These studies assess the effectiveness of interventions such as needle and syringe programs, opioid substitution therapy, and antiviral treatment in reducing HCV transmission. This topic relates to harm reduction by addressing the prevention of HCV infection among PWID.
Drug use and harm reduction interventions	Articles examining various harm reduction interventions for drug users, including overdose prevention programs, syringe exchange programs, addiction interventions, and risk reduction strategies. These studies assess the effectiveness of different interventions in reducing harm associated with drug use. This topic directly relates to harm reduction by focusing on interventions aimed at minimizing the negative consequences of drug use.
Heroin and opioid use	Articles investigating the patterns, perceptions, and risks associated with heroin and opioid use. These studies often explore issues such as adulteration of heroin, transition to illicit drug injection, opioid overdose deaths, and access to opioid substitution therapy. This topic provides insights into the specific challenges and risks associated with heroin and opioid use, which are central to harm reduction efforts.
Opioid overdose and mortality	Articles examining opioid overdose rates, mortality, and the impact of interventions such as naloxone distribution, overdose education, and addiction treatment programs on reducing overdose deaths. This topic directly addresses harm reduction by focusing on strategies to prevent opioid overdose fatalities.
Injection drug use and risk behaviors	Articles investigating risk behaviors associated with injection drug use, including high-risk sexual behaviors, sharing of drug injection equipment, and the spread of infectious diseases. These studies often assess the impact of harm reduction programs on reducing risky behaviors and promoting safer injection practices. This topic highlights the importance of harm reduction interventions in minimizing risks related to injection drug use.
Policies and strategies for harm reduction	Articles discussing policies, legal changes, and strategies aimed at increasing access to harm reduction interventions such as naloxone, syringe exchange programs, and opioid substitution therapy. These studies examine the impact of different policy approaches on reducing drug-related harms and improving public health outcomes. This topic addresses harm reduction from a broader policy and implementation perspective.
Peer education and support interventions	Articles evaluating the effectiveness of peer-education interventions and support programs in reducing risk behaviors, improving access to care, and enhancing adherence to HIV medications among injection drug users. These studies highlight the value of peer-based approaches in harm reduction efforts and promoting positive health outcomes among drug users.
Methadone treatment and mortality	Articles assessing the impact of methadone treatment, an opioid substitution therapy, on mortality rates among heroin users recognize the effectiveness of opioid substitution therapy in reducing harms associated with heroin use. These studies contribute to harm reduction by providing evidence of the benefits of methadone treatment in improving health outcomes and reducing mortality rates.

**Table 2:** Research topics in the top 50 recently published articles on harm reduction related to injectable drug use

Research Topic	Description and Relation to Harm Reduction
Opioid Overdose Prevention	Articles focusing on strategies, interventions, and policies aimed at preventing opioid overdose, including naloxone distribution programs, overdose risk mitigation, overdose prevention training, and opioid overdose counseling. These topics are directly related to harm reduction efforts by addressing the immediate risks associated with opioid use.
HIV Prevention and Service Delivery for People Who Inject Drugs	Articles examining the effectiveness of HIV prevention interventions, such as pre-exposure prophylaxis (PrEP), testing, linkage to care, and engagement in HIV services specifically tailored for people who inject drugs. These topics are relevant to harm reduction as they aim to reduce the transmission of HIV among this population and improve access to healthcare services.
Hepatitis C Virus (HCV) Prevention and Treatment	Articles focusing on the prevention, testing, treatment, and outcomes of hepatitis C virus among people who inject drugs. These topics are crucial for harm reduction efforts as they address the high prevalence of HCV among this population and aim to reduce the transmission and improve the health outcomes related to HCV infection.
Stigma Reduction and Attitudes toward Opioid Use Disorder	Articles exploring the stigma surrounding opioid use disorder and attitudes toward harm reduction strategies. These topics are relevant to harm reduction by addressing the social barriers and negative perceptions associated with opioid use, which can hinder access to care and harm reduction services.
COVID-19 Pandemic and its Impact on People Who Inject Drugs	Articles examining the impact of the COVID-19 pandemic on people who inject drugs, including access to harm reduction services, vaccination uptake, and changes in drug use behaviors. These topics highlight the importance of adapting harm reduction efforts in response to public health emergencies.
Social Network Analysis and Modeling of HIV and Drug Use	Articles utilizing social network analysis and mathematical modeling to understand HIV transmission dynamics among people who inject drugs. These topics contribute to harm reduction efforts by providing insights into the patterns of HIV transmission and informing the development of targeted interventions.
Needle and Syringe Programs	Articles evaluating the effectiveness and outcomes of needle and syringe exchange programs as a harm reduction strategy. These topics focus on providing access to sterile injecting equipment and facilitating safer drug use practices to reduce the risks of blood-borne infections.
Overdose Risk and Fentanyl-related Concerns	Articles investigating the risks associated with fentanyl use, multiple naloxone administration for overdose reversal, and concerns related to fentanyl overdose among people who inject drugs. These topics address the evolving landscape of drug use and its impact on harm reduction efforts.
Other Topics	The remaining articles cover a variety of topics, including occupational roles and risks of peer educators, depression and secondary HIV transmission, ED-based referral tools for HIV/HCV and overdose prevention, and gender-based violence prevention among key populations. Although these topics may not directly relate to harm reduction, they contribute to the broader understanding of the intersecting issues and challenges faced by people who inject drugs.

1. Time period: The research topics in the 50 most cited articles appear to cover a more extensive time span, possibly ranging from earlier years up to the present. On the other hand, the research topics in the 50 most recently published articles are focused on current and emerging issues in the field, likely reflecting more recent developments in harm reduction efforts.
2. Focus on naloxone distribution: Naloxone distribution and overdose prevention emerge as a prominent research topic in the highly cited articles, with 9 articles dedicated to this subject. In contrast, while opioid overdose prevention is still a prevalent topic in the recently published articles, it is not as dominant as in the highly cited articles.
3. HIV and Hepatitis C transmission: In the highly cited articles, HIV transmission and injection drug use, as well as hepatitis C transmission and injection drug use, are prominent research topics, with 8 and 7 articles, respectively. While HIV prevention and service delivery for people who inject drugs and hepatitis C virus (HCV) prevention and treatment are also relevant topics in the recently published articles, they seem to have slightly less emphasis compared to the older publications.
4. COVID-19 pandemic impact: The impact of the COVID-19 pandemic on people who inject drugs emerges as a research topic in the recently published articles, which is not present in the highly cited articles. This indicates a focus on understanding and adapting harm reduction efforts during public health emergencies.
5. Social network analysis and mathematical modeling: The use of social network analysis and mathematical modeling to understand HIV transmission dynamics among people who inject drugs is more pronounced in the recently published articles, whereas it is not as prominently featured in the highly cited articles.
6. Fentanyl-related concerns: Articles investigating fentanyl-related concerns and risks associated with fentanyl use are notable research topics in the recently published articles, which may reflect the growing concern over fentanyl’s impact on opioid overdose deaths.
7. Stigma reduction and attitudes: Research topics related to stigma reduction and attitudes toward opioid use disorder appear in the recently published articles, highlighting the importance of addressing social barriers and negative perceptions that can hinder harm reduction efforts.
8. Needle and Syringe Programs: While needle and syringe programs are still relevant in both sets of articles, they appear to be more explicitly explored in the recently published articles, indicating ongoing interest in this harm reduction strategy.

Overall, the research topics in both sets of articles share a common focus on harm reduction strategies and interventions. However, the more recent articles seem to emphasize emerging challenges, such as fentanyl-related risks and the impact of the COVID-19 pandemic, while the highly cited articles encompass a broader time frame and may have laid the foundation for some of the harm reduction efforts seen in the more recent publications.

## Discussion

The current study analyzed and mapped the research activity on harm reduction in the context of IDU. The analysis showed steady growth over time, which can be attributed to the increased spread of HIV and HCV among people who inject drugs. This public health crisis highlighted the urgent need for effective harm reduction interventions, such as needle and syringe exchange programs, to prevent the transmission of these blood-borne infections. The increasing research focus can also be attributed to the immediate need for evidence-based strategies to address the spread of HIV and other infectious diseases transferred through contaminated needles [21–25]. The steeper growth in research from 2015 to 2022 may reflect the increased demand for evidence to inform the expansion and improvement of these interventions [9, 26–28]. In recent years, there has been a growing recognition of the social determinants of drug use and related health disparities. This broader understanding acknowledges that substance use is influenced by complex social, economic, and structural factors [29–31]. Consequently, research on harm reduction interventions has expanded to examine the intersecting issues of poverty, inequality, stigma, mental health, and access to healthcare. This multidimensional approach has likely contributed to the increased research interest and the steeper growth observed in recent years. Overall, the growth pattern in research on harm reduction interventions in the context of IDU reflects a combination of factors, including increased recognition of the importance of harm reduction, the impact of HIV and HCV epidemics, policy and program expansions, evolving drug use landscapes, and a broader understanding of social determinants. This growth signifies the ongoing commitment to addressing the complex challenges faced by individuals who inject drugs and the continuous efforts to develop effective interventions that prioritize harm reduction and public health.

The current study showed that research activity on harm reduction in the context of IDU was mainly limited to high-income, English-speaking countries. The reasons for research activity in the field of harm reduction interventions in different countries are multifaceted, involving factors such as the prevalence of IDU, research capacities, government support, international collaboration, and unique socioeconomic and cultural contexts [32–35]. It is a combination of these factors that contribute to the research focus in specific countries. The fact that the *Harm Reduction* journal and the *International Journal of Drug Policy* rank highly and contribute to approximately one third of the research output on harm reduction interventions in the context of IDU indicates their significant role and influence in this area of study. This research activity can be interpreted as a reflection of the growing recognition of the importance of harm reduction approaches in addressing the complex issues associated with IDU. These journals’ focus on harm reduction aligns with the broader shift in drug policy and public health approaches towards a more compassionate and evidence-based approach to drug use. Harm reduction recognizes that complete eradication of drug use may not be feasible, and instead aims to minimize the harms and risks associated with drug use for both individuals and communities. The research output in this field may be driven by several factors. Firstly, there is a pressing need to develop effective interventions to address the increasing rates of IDU and related health risks, including HIV and hepatitis C transmission. Harm reduction interventions, such as needle and syringe programs, naloxone distribution, and opioid agonist treatment, have shown to be effective in reducing these harms [36, 37]. Secondly, the research activity may be driven by the urgency of the opioid crisis and its devastating impact on individuals, families, and communities [38–40]. The opioid crisis has heightened public awareness and policymakers’ attention to the need for evidence-based strategies, such as harm reduction, to address the escalating overdose rates and associated mortality. Furthermore, the research activity may be influenced by the recognition that criminalization and punitive approaches have proven ineffective in reducing drug-related harms and have contributed to the stigmatization and marginalization of drug users. Harm reduction provides an alternative framework that emphasizes public health, human rights, and social justice, which resonates with researchers, policymakers, and advocates seeking more humane and effective solutions [41–43]. The research output in these journals reflects the interdisciplinary nature of harm reduction, drawing on fields such as public health, medicine, social sciences, and policy studies. It highlights the collaborative efforts of researchers, practitioners, and policymakers to generate evidence, evaluate interventions, and inform policy and practice. In summary, the prominence of the *Harm Reduction* journal and the *International Journal of Drug Policy* in the research output on harm reduction interventions in the context of IDU indicates the significance and relevance of this research area. It reflects the growing recognition of harm reduction as a compassionate and evidence-based approach to addressing the complex challenges associated with IDU, particularly in the context of the opioid crisis and the need for effective interventions to reduce harm and improve public health outcomes.

The major common research points between the highly cited articles and the recently published articles include a focus on harm reduction strategies, interventions, and policies related to opioid overdose prevention, HIV prevention and service delivery for people who inject drugs, HCV testing and treatment, and needle and syringe programs. These areas of research demonstrate the ongoing efforts to address the complex challenges associated with substance use, harm reduction, and public health. Both sets of highly cited and recently published articles share a common goal of advancing harm reduction efforts and improving the health and well-being of individuals who inject drugs. However, there were certain important differences in the two sets that can be attributed to the time difference. The highly cited articles cover a broader time span, including earlier years up to the present, whereas the recently published articles are focused on current and emerging issues. This difference reflects the evolving nature of research interests and the changing landscape of harm reduction efforts over time. Naloxone distribution and overdose prevention appear as a prominent research topic in the highly cited articles [44, 45]. This likely reflects the increasing recognition of naloxone as a life-saving intervention for opioid overdoses and the efforts to expand access to this medication. In the recently published articles, while naloxone distribution and overdose prevention remain relevant, they may not be as dominant due to the maturation of research in this area. The highly cited articles give considerable attention to HIV and hepatitis C transmission among people who inject drugs, indicating the historical significance of these issues and the focus on preventing the spread of bloodborne infections [46–48]. While HIV prevention and HCV prevention and treatment are still prominent research topics in the recently published articles, they may have slightly less emphasis compared to the older publications, reflecting advancements and progress made in these areas. The recently published articles include research on the impact of the COVID-19 pandemic on people who inject drugs and harm reduction services [49–51]. This research topic is not present in the highly cited articles, indicating a response to the ongoing public health crisis and the need to understand and adapt harm reduction efforts during such emergencies. The use of social network analysis and mathematical modeling to study HIV transmission dynamics among people who inject drugs is more pronounced in the recently published articles [52–55]. This reflects the increasing recognition of the role of social networks and complex systems in understanding disease transmission and designing targeted interventions. Articles investigating fentanyl-related concerns and risks associated with fentanyl use emerge as notable research topics in the recently published articles [56, 57]. This reflects the growing concern over fentanyl’s role in the opioid crisis and its impact on opioid overdose deaths, indicating a need for research to address this specific challenge. The recently published articles include research topics related to stigma reduction and attitudes toward opioid use disorder [58, 59]. This highlights the recognition that addressing stigma and negative perceptions is crucial for effective harm reduction strategies and improving access to services. While needle and syringe programs remain relevant in both sets of articles, they appear to be more explicitly explored in the recently published articles. This may reflect a continued interest in evaluating the effectiveness and impact of these harm reduction strategies and exploring their implementation in different contexts.

### Research/knowledge gaps

Based on the research topics and mapping of keywords and items in titles and abstracts of the retrieved articles, several research gaps can be identified in the context of harm reduction for injecting drug users (IDU). Firstly, there is a limited focus on low- and middle-income countries, as the current study reveals that research activity on harm reduction interventions is primarily concentrated in high-income, English-speaking countries. More research is needed in low- and middle-income regions where IDU and associated harms may be prevalent but inadequately addressed. Secondly, with the evolving landscape of drug use, including the emergence of new substances like synthetic opioids such as fentanyl, more research is necessary to understand their impact on harm reduction interventions and to develop effective strategies to mitigate their potential harms. Additionally, a comprehensive understanding of social determinants is crucial. While recent research recognizes the influence of social determinants on drug use and related health disparities, further investigation is required to better understand and address the complex interactions of poverty, inequality, stigma, mental health, and access to healthcare in harm reduction contexts. There is also a need for rigorous evaluation studies to assess the long-term impact and cost-effectiveness of harm reduction interventions, such as needle and syringe programs, naloxone distribution, and opioid agonist treatment, in various settings. Furthermore, research gaps exist in understanding the unique needs and challenges faced by marginalized and vulnerable populations, including women who inject drugs, LGBTQ + communities, and racial or ethnic minorities. Tailored harm reduction interventions that address these groups’ specific needs are essential for equitable access to services. There is also a lack of longitudinal studies tracking the implementation and effectiveness of harm reduction interventions over time. Long-term studies are necessary to monitor trends, evaluate progress, and identify areas for improvement. Moreover, comparative studies across different harm reduction approaches and models can help identify the most effective strategies for specific populations and settings. Comparative effectiveness studies can contribute to evidence-based policymaking and program implementation. Lastly, further research is needed to understand the barriers and facilitators of implementing harm reduction programs and policies on a broader scale. Identifying successful implementation strategies can support the uptake of evidence-based interventions in various contexts. Addressing these research gaps can enhance the effectiveness and impact of harm reduction interventions for IDU, contributing to improved public health outcomes and reduced harm for individuals and communities affected by drug use.

### Limitations

The use of Scopus as the primary data source may result in the exclusion of articles from other databases or gray literature sources. This could introduce a potential bias in the findings. The study focused on articles published in English, which may lead to the exclusion of relevant non-English publications. The analysis of recently published articles was based on a specific time frame, and therefore, the identified emerging topics and future directions may not capture the entire spectrum of ongoing research in the field. The inclusion and exclusion criteria used for article screening may introduce some degree of selection bias, potentially omitting relevant studies. The absence of false-positive and false-negative results was based on several validity tests that ensures the comprehensiveness of research strategy but no guarantee of the accuracy of the retrieved articles remained less than perfect. This should not be considered a major drawback in a study that focus on research patterns and trends rather than on a specific research question as in the case of systematic review. Finally, the research gaps provided in the current study was based on research mapping rather than screening the articles themselves. Therefore, these research gaps remain an overall picture of the research status rather than specific detailed research gaps based on the content analysis of the articles.

## Conclusions

In conclusion, this study has provided valuable insights into the research activity on harm reduction in the context of IDU. The findings demonstrate steady growth in research over time, driven by various factors. The increasing recognition of the importance of harm reduction approaches, the impact of HIV and HCV epidemics, the development of policies and programs, the evolving drug use landscape, and a broader understanding of social determinants have all contributed to the growth of research in this field. The study also revealed that research activity is primarily concentrated in high-income, English-speaking countries, highlighting the need for more research in low- and middle-income countries. The prominence of journals such as the Harm Reduction journal and the International Journal of Drug Policy indicates their significant role in shaping this field of study and aligns with the broader shift towards evidence-based and compassionate approaches to drug use. Common research areas between highly cited and recently published articles include opioid overdose prevention, HIV prevention, HCV testing and treatment, and needle and syringe programs, reflecting the ongoing commitment to harm reduction efforts. However, recent publications have focused more on emerging challenges such as the impact of the COVID-19 pandemic, fentanyl-related risks, stigma reduction, and attitudes toward opioid use disorder. The identified research gaps call for further research in low- and middle-income countries, addressing new and emerging substances, comprehensive understanding of social determinants, evaluating program effectiveness, including diverse populations, conducting longitudinal and comparative studies, and investigating implementation strategies. Addressing these gaps will contribute to the development of more effective interventions and policies that prioritize harm reduction and public health, ultimately improving the well-being of individuals who inject drugs and the communities they belong to.

### Electronic supplementary material

Below is the link to the electronic supplementary material.

**Supplementary Material 1:Supplement 1**. Keywords used in the search strategy

**Supplementary Material 2:Supplement 2**. Flow diagram for the number of retrieved articles

**Supplementary Material 3:Supplement 3**. Top 50 cited articles

**Supplementary Material 4:Supplement 4**. Top 50 recently published articles

## Data Availability

All data presented in this manuscript are available on the Scopus database (www.scopus.com) using the search query listed in the methodology section.

## References

[1] Battle DE (2013). Diagnostic and statistical Manual of Mental disorders (DSM). Codas.

[2] First MB. Diagnostic and statistical manual of mental disorders, 5th edition, and clinical utility. J Nerv Ment Dis. 2013;201(9):727-9. 10.1097/NMD.0b013e3182a2168a. 10.1097/NMD.0b013e3182a2168a 23995026

[3] Kamali M, Tajadini H, Mehrabani M, Moghadari M (2020). Consequences of opioid abuse and their treatments in Persian Medicine: a review study. Addict Health.

[4] Macdonald M (2013). Women prisoners, mental health, violence and abuse. Int J Law Psychiatry.

[5] Worls Health Organization (WHO). Substance abuse. Accessed June, 29. 2023. https://www.afro.who.int/health-topics/substance-abuse.

[6] Degenhardt L, Peacock A, Colledge S, Leung J, Grebely J, Vickerman P (2017). Global prevalence of injecting drug use and sociodemographic characteristics and prevalence of HIV, HBV, and HCV in people who inject drugs: a multistage systematic review. Lancet Glob Health.

[7] Larney S, Peacock A, Mathers BM, Hickman M, Degenhardt L (2017). A systematic review of injecting-related injury and disease among people who inject drugs. Drug Alcohol Depend.

[8] (UNODC) UNOoDaC. World Drug Report 2022. 2022. Accessed June 29, 2023. https://www.unodc.org/unodc/data-and-analysis/world-drug-report-2022.html.

[9] Levengood TW, Yoon GH, Davoust MJ, Ogden SN, Marshall BDL, Cahill SR (2021). Supervised Injection facilities as Harm reduction: a systematic review. Am J Prev Med.

[10] Des Jarlais DC (2017). Harm reduction in the USA: the research perspective and an archive to David Purchase. Harm Reduct J.

[11] Krawczyk N, Fawole A, Yang J, Tofighi B (2021). Early innovations in opioid use disorder treatment and harm reduction during the COVID-19 pandemic: a scoping review. Addict Sci Clin Pract.

[12] Hawk KF, Vaca FE, D’Onofrio G (2015). Reducing fatal opioid overdose: Prevention, Treatment and Harm reduction strategies. Yale J Biol Med.

[13] Gugala E, Briggs O, Moczygemba LR, Brown CM, Hill LG (2022). Opioid harm reduction: a scoping review of physician and system-level gaps in knowledge, education, and practice. Subst Abus.

[14] Kerman N, Polillo A, Bardwell G, Gran-Ruaz S, Savage C, Felteau C (2021). Harm reduction outcomes and practices in Housing First: a mixed-methods systematic review. Drug Alcohol Depend.

[15] Al-Hamdani M, Manly E (2022). Harm reduction in tobacco control: where do we draw the line?. J Public Health Policy.

[16] Dunne RB (2018). Prescribing naloxone for opioid overdose intervention. Pain Manag.

[17] Hser YI, Liang D, Lan YC, Vicknasingam BK, Chakrabarti A (2016). Drug abuse, HIV, and HCV in Asian countries. J Neuroimmune Pharmacol.

[18] Denzin NK. The landscape of qualitative research. Sage; 2008.

[19] Sweileh WM, Wickramage K, Pottie K, Hui C, Roberts B, Sawalha AF (2018). Bibliometric analysis of global migration health research in peer-reviewed literature (2000–2016). BMC Public Health.

[20] van Eck NJ, Waltman L (2010). Software survey: VOSviewer, a computer program for bibliometric mapping. Scientometrics.

[21] Marks LR, Nolan NS, Liang SY, Durkin MJ, Weimer MB (2022). Infectious complications of Injection Drug Use. Med Clin North Am.

[22] Taylor JL, Johnson S, Cruz R, Gray JR, Schiff D, Bagley SM (2021). Integrating harm reduction into Outpatient Opioid Use Disorder Treatment settings: Harm Reduction in Outpatient Addiction Treatment. J Gen Intern Med.

[23] Bao YP, Liu ZM (2009). Systematic review of HIV and HCV infection among drug users in China. Int J STD AIDS.

[24] Stöver H, Dichtl A, Schäffer D, Grabski M (2023). HIV and HCV among drug users and people living in prisons in Germany 2022: WHO elimination targets as reflected in practice. Harm Reduct J.

[25] Getahun H, Gunneberg C, Sculier D, Verster A, Raviglione M (2012). Tuberculosis and HIV in people who inject drugs: evidence for action for tuberculosis, HIV, prison and harm reduction services. Curr Opin HIV AIDS.

[26] Page JB (1997). Needle exchange and reduction of harm: an anthropological view. Med Anthropol.

[27] Belisle LA, Solano-Patricio EDC (2021). Harm reduction: a public health approach to prison drug use. Int J Prison Health.

[28] Wilkinson R, Hines L, Holland A, Mandal S, Phipps E (2020). Rapid evidence review of harm reduction interventions and messaging for people who inject drugs during pandemic events: implications for the ongoing COVID-19 response. Harm Reduct J.

[29] Dengo-Baloi L, Boothe M, Semá Baltazar C, Sathane I, Langa DC, Condula M (2020). Access to and use of health and social services among people who inject drugs in two urban areas of Mozambique, 2014: qualitative results from a formative assessment. BMC Public Health.

[30] Rudolph AE, Young AM, Havens JR (2017). Examining the Social Context of Injection Drug Use: Social Proximity to persons who inject drugs Versus Geographic Proximity to persons who inject drugs. Am J Epidemiol.

[31] Mburu G, Limmer M, Holland P (2019). Role of boyfriends and intimate sexual partners in the initiation and maintenance of injecting drug use among women in coastal Kenya. Addict Behav.

[32] Bui H, Zablotska-Manos I, Hammoud M, Jin F, Lea T, Bourne A (2018). Prevalence and correlates of recent injecting drug use among gay and bisexual men in Australia: results from the FLUX study. Int J Drug Policy.

[33] Argento E, Chettiar J, Nguyen P, Montaner J, Shannon K (2015). Prevalence and correlates of nonmedical prescription opioid use among a cohort of sex workers in Vancouver, Canada. Int J Drug Policy.

[34] Sherman SG, Cheng Y, Kral AH (2007). Prevalence and correlates of opiate overdose among young injection drug users in a large U.S. city. Drug Alcohol Depend.

[35] Johnston LG, Corceal S (2013). Unexpectedly high injection drug use, HIV and Hepatitis C prevalence among female sex workers in the Republic of Mauritius. AIDS Behav.

[36] Palmateer N, Hamill V, Bergenstrom A, Bloomfield H, Gordon L, Stone J (2022). Interventions to prevent HIV and Hepatitis C among people who inject drugs: latest evidence of effectiveness from a systematic review (2011 to 2020). Int J Drug Policy.

[37] MacArthur GJ, van Velzen E, Palmateer N, Kimber J, Pharris A, Hope V (2014). Interventions to prevent HIV and Hepatitis C in people who inject drugs: a review of reviews to assess evidence of effectiveness. Int J Drug Policy.

[38] Coussens NP, Sittampalam GS, Jonson SG, Hall MD, Gorby HE, Tamiz AP (2019). The Opioid Crisis and the future of Addiction and Pain therapeutics. J Pharmacol Exp Ther.

[39] Upp LA, Waljee JF (2020). The opioid epidemic. Clin Plast Surg.

[40] Chisholm-Burns MA, Spivey CA, Sherwin E, Wheeler J, Hohmeier K (2019). The opioid crisis: origins, trends, policies, and the roles of pharmacists. Am J Health Syst Pharm.

[41] Amèrica-Simms L (2011). Global: commission on drug policy declares drug war a failure, urges reforms. HIV AIDS Policy Law Rev.

[42] Frank D (2020). Methadone maintenance treatment is swapping one drug for another, and that’s why it works: towards a treatment-based critique of the war on drugs. Int J Drug Policy.

[43] Iacobucci G (2016). War on drugs has harmed public health and human rights, finds new analysis. BMJ.

[44] Doe-Simkins M, Quinn E, Xuan Z, Sorensen-Alawad A, Hackman H, Ozonoff A, et al. Overdose rescues by trained and untrained participants and change in opioid use among substance-using participants in overdose education and naloxone distribution programs: a retrospective cohort study. BMC Public Health. 2014;14(1). 10.1186/1471-2458-14-297. 10.1186/1471-2458-14-297 PMC4004504 24684801

[45] Green TC, Heimer R, Grau LE (2008). Distinguishing signs of opioid overdose and indication for naloxone: an evaluation of six overdose training and naloxone distribution programs in the United States. Addiction.

[46] Bruneau J, Lamothe F, Franco E, Lachance N, Désy M, Soto J (1997). High rates of HIV infection among injection drug users participating in needle exchange programs in Montreal: results of a cohort study. Am J Epidemiol.

[47] Schlechter MT, Strathdee SA, Cornelisse PGA, Currie S, Patrick DM, Rekart ML (1999). Do needle exchange programmes increase the spread of HIV among injection drug users? An investigation of the Vancouver outbreak. AIDS.

[48] Garfein RS, Golub ET, Greenberg AE, Hagan H, Hanson DL, Hudson SM, et al. A peer-education intervention to reduce injection risk behaviors for HIV and Hepatitis C virus infection in young injection drug users. AIDS. 2007;21(14):1923–32. 10.1097/QAD.0b013e32823f9066. 10.1097/QAD.0b013e32823f9066 17721100

[49] Curado A, Nogueira PJ, Virgolino A, Santa Maria J, Mendão L, Furtado C, et al. Hepatitis C antibody prevalence and behavioral correlates in people who inject drugs attending harm reduction services in Lisbon, Portugal. Front Public Health. 2022;10. 10.3389/fpubh.2022.952909. 10.3389/fpubh.2022.952909 PMC9445135 36081480

[50] Knaub RJ, Evans J, Yang C, Roura R, McGinn T, Verschoore B, et al. A pilot study of a mixed-method approach to design an ED-based peer mHealth referral tool for HIV/HCV and opioid overdose prevention services. Drug Alcohol Depend. 2022;238. 10.1016/j.drugalcdep.2022.109585. 10.1016/j.drugalcdep.2022.109585 PMC9620482 35926299

[51] Jarlais DD, Bobashev G, Feelemyer J, McKnight C. Modeling HIV transmission among persons who inject drugs (PWID) at the end of the HIV Epidemic and during the COVID-19 pandemic. Drug Alcohol Depend. 2022;238. 10.1016/j.drugalcdep.2022.109573. 10.1016/j.drugalcdep.2022.109573 PMC9278993 35926301

[52] Singleton AL, Marshall BDL, Bessey S, Harrison MT, Galvani AP, Yedinak JL, et al. Network structure and rapid HIV transmission among people who inject drugs: a simulation-based analysis. Epidemics. 2021;34. 10.1016/j.epidem.2020.100426. 10.1016/j.epidem.2020.100426 PMC7940592 33341667

[53] Cousien A, Tran VC, Deuffic-Burban S, Jauffret-Roustide M, Dhersin JS, Yazdanpanah Y (2015). Dynamic modelling of hepatitis C virus transmission among people who inject drugs: a methodological review. J Viral Hepat.

[54] Mumtaz GR, Awad SF, Feizzadeh A, Weiss HA, Abu-Raddad LJ (2018). HIV incidence among people who inject drugs in the Middle East and North Africa: mathematical modelling analysis. J Int AIDS Soc.

[55] Corson S, Greenhalgh D, Taylor A, Palmateer N, Goldberg D, Hutchinson S (2013). Modelling the prevalence of HCV amongst people who inject drugs: an investigation into the risks associated with injecting paraphernalia sharing. Drug Alcohol Depend.

[56] Karamouzian M, Dohoo C, Forsting S, McNeil R, Kerr T, Lysyshyn M (2018). Evaluation of a fentanyl drug checking service for clients of a supervised injection facility, Vancouver, Canada. Harm Reduct J.

[57] Coffin PO, Maya S, Kahn JG. Modeling of overdose and naloxone distribution in the setting of fentanyl compared to heroin. Drug Alcohol Depend. 2022;236. 10.1016/j.drugalcdep.2022.109478. 10.1016/j.drugalcdep.2022.109478 PMC9235402 35588609

[58] Bascou NA, Haslund-Gourley B, Amber-Monta K, Samson K, Goss N, Meredith D, et al. Reducing the stigma surrounding opioid use disorder: evaluating an opioid overdose prevention training program applied to a diverse population. Harm Reduct J. 2022;19(1). 10.1186/s12954-022-00589-6. 10.1186/s12954-022-00589-6 PMC8761384 35034649

[59] Gibson K, Hutton F (2021). Women who inject drugs (WWID): stigma, gender and barriers to Needle Exchange Programmes (NEPs). Contemp Drug Probl.

