# Supplementary material for: Analysis and mapping of harm reduction research in the context of injectable drug use: identifying research hotspots, gaps and future directions
Source: Harm Reduct J. 2024 Jul 10;21:131. doi: 10.1186/s12954-024-01048-0 (PMC11234666; doi:10.1186/s12954-024-01048-0)
Supplement: Supplementary file 1 — Supplementary Material 1:Supplement 1. Keywords used in the search strategy [file 12954_2024_1048_MOESM1_ESM.docx]

**Analysis and mapping of harm reduction research in the context of injectable drug use: Identifying research hotspots, gaps and future directions**

**Supplement 1**

**Keywords, inclusion and exclusion criteria, and search strategy**

| **Concept** | **Keywords** |
| --- | --- |
| **Harm reduction interventions/strategies/initiatives/policies/approaches/ services/treatment/ etc** | "collaboration with community organizations" or "family and social network involvement" or "supportive housing" or "employment and vocational support" or "education and awareness" or "policy interventions" or "treatment and rehabilitation" or "media and advertising regulations" or "research and monitoring" or "prevent* blood borne infect*" or "harm reduction" or "harm minimization" or "risk reduction" or "damage reduction" or "health promotion" or "safer use" or "safety-focused approaches" or "minimizing adverse consequences" or "public health approach" or "health-centered interventions" or "prevention of harm" or "behavior-based interventions" or "reduction of negative outcomes" or "mitigation of risks" or "safety strategies" or "health-based interventions" or "needle exchange program*" or "safer injection practice*" or "overdose prevention" or "safe substance use" or "substance use treatment alternatives" or "syringe access program*" or "safer drug use practice*" or "syringe exchange program" or "hiv transmission" or "virus transmission" or "condom use" or ("blood borne infect*" and prevent* ) or "needle and syringe program*" or "needle distribution program*" or "safe needle program*" or "harm reduction services" or "opioid substitution therapy" or "medication-assisted treatment" or "opioid detoxification programs" or "education and awareness campaign*" or "substance abuse prevention campaign*" or "harm reduction education" or "drug education programs" or "public awareness initiatives" or "information campaign*" or "safer sex practices" or "harm reduction in sexual behavior" or "safe sex education" or "condom use promotion" or "barrier method education" or "preventing sexually transmitted" or "harm reduction drug testing" or "naloxone distribution" or "naloxone access programs" or "overdose reversal medication distribution" or "narcan distribution" or "opioid overdose rescue kits" or "safe injection sites" or "supervised consumption facilit*" or "harm reduction injection facilit*" or "medically supervised drug consumption site*" or "overdose prevention centers" or "supervised injection services" or "safe drug use facilit*" or "street outreach service*" or "mobile harm reduction program*" or "outreach services for people who use drugs" or "harm reduction outreach activities" or "peer support program*" or "peers in recovery programs" or "harm reduction peer support" or "peer mentoring for substance abuse" or "support networks of individuals with lived experience" or "peer-based harm reduction services" or "overdose prevention education" or "overdose response training" or "naloxone training program*" or "opioid overdose education" or "recognizing and responding to overdose*" or "overdose prevention initiative*" or "access to healthcare service*" or "healthcare for people who use drugs" or "substance abuse treatment access" or "integrated healthcare for substance use disorder*" or "addiction treatment service*" or "medical service* for substance abuser*" or "harm reduction policies and advocacy" or "public health campaign*" or "harm reduction in prison*" or "harm reduction psychotherapy" or "safe disposal of drug paraphernalia" or "overdose prevention" or "naloxone distribution" or "opioid overdose reversal" or "safe injection site*" or "supervised injection facilit*" or "managed drug consumption site*" |
| **Injectable drugs** | opioid* or heroin or "injectable drug" or "inject* drugs" or (“inject" and stimulant*) or "needle drug use" or (inject and cocaine) or (inject and amphetamine) or (inject* and drugs) |

| **Inclusion criteria** | **Exclusion Criteria** |
| --- | --- |
| Peer-reviewed research articles | Books, book chapters, editorials, notes, letters, conference proceedings, and reviews |
| All articles published after 1980 | Articles published in 2023 |
| All Articles published in English | Articles published in non-English language |
| All articles with keywords related to harm reduction and injectable drug use in titles and abstracts |  |
