# Supplementary material for: Analysis and mapping of harm reduction research in the context of injectable drug use: identifying research hotspots, gaps and future directions
Source: Harm Reduct J. 2024 Jul 10;21:131. doi: 10.1186/s12954-024-01048-0 (PMC11234666; doi:10.1186/s12954-024-01048-0)
Supplement: Supplementary file 2 — Supplementary Material 2:Supplement 2. Flow diagram for the number of retrieved articles [file 12954_2024_1048_MOESM2_ESM.docx]

**Supplement 2:** Adapted PRISMA 2020 framework of the search strategy. Source: Adapted from Page et al. (Page et al., 2021)

**Identification of records via Scopus database**

Records identified from the initial search in Scopus database using the listed keywords.

(n = 1463):

No duplicates found

(n=0)

**Identification**

Records published after December 31, 2022, editorials, notes, errata, books, book chapters, conference papers were excluded.

(n = 426)

Records filtered from Scopus database searching

(n = 1463)

False positive records excluded* (n = 66)

Records sought for analysis

(n = 1037)

**Screening**

Validated records

(n = 971)

Records eligible for final analysis

(n=971)

**Included**
